# PERK Regulates the Sensitivity of Hepatocellular Carcinoma Cells to High-LET Carbon Ions via either Apoptosis or Ferroptosis

**DOI:** 10.7150/jca.61622

**Published:** 2022-01-01

**Authors:** Xiaogang Zheng, Bingtao Liu, Xiongxiong Liu, Ping Li, Pengcheng Zhang, Fei Ye, Ting Zhao, Yanbei Kuang, Weiqiang Chen, Xiaodong Jin, Qiang Li

**Affiliations:** 1 Institute of Modern Physics, Chinese Academy of Sciences, Lanzhou 730000, China.; 2 Key Laboratory of Heavy Ion Radiation Biology and Medicine, Chinese Academy of Sciences, Lanzhou 730000, China.; 3 Key Laboratory of Basic Research on Heavy Ion Radiation Application in Medicine, Gansu Province, Lanzhou 730000, China.; 4 University of Chinese Academy of Sciences, Beijing 100049, China.

**Keywords:** PERK, carbon ion, ER stress, autophagy, ferroptosis

## Abstract

PERK is one of the transmembrane sensors of unfolded protein response (UPR) triggered by ER stress. In this study, we evaluated the role of PERK in the sensitivity of hepatocellular carcinoma (HCC) cells to high linear energy transfer (LET) carbon ions (CI). We found that CI irradiation could induce ER stress in HCC cells. On the one hand, PERK promoted autophagy via regulating ATF4 expression; on the other hand, PERK regulated p53 expression, and the latter either induced autophagy through up-regulating DRAM, or directly promoting apoptosis through the mitochondrial pathway or facilitating ferroptosis via down-regulating *SLC7A11* (the extrinsic pathway), but independent of GPX4 (the intrinsic pathway). These factors jointly determined the sensitivity of HCC cells to high-LET CI radiation. Inhibiting *TP53* directly increased cellular radioresistance definitely. Moreover, the death of HepG2 (*TP53* wild type) cells induced by high-LET CI irradiation combined with sorafenib treatment might be caused by a mixed-type regulated cell death (RCD) including both apoptosis and ferroptosis, suggesting that apoptosis and ferroptosis are synergetic cell death modes regulated by *TP53*, which is one of the reasons why the sensitivity of HepG2 cells is higher than that of Hep3B (*TP53* null type) and PLC/PRF5 (*TP53* mutated type) cells. Therefore, our work might shed light on the potential therapeutic implication of CI radiotherapy combined with PERK targeted clinical drugs to implement personalized and precise treatment of HCCs.

## Introduction

Ionizing radiation brings about damage to biological macromolecules such as DNA and proteins, as well as organelles such as mitochondria and endoplasmic reticulum (ER) through direct and indirect effects, causing cell cycle arrest, autophagy and ER stress, and eventually leading to cell death 1. The radiobiological characteristics make high linear energy transfer (LET) carbon ions (CI) possess great advantages in the treatment of tumors resistant to conventional radiations 2. At present, the main treatment modalities of HCC are surgery, liver transplantation, percutaneous or arterial intervention, small molecule multi-kinase inhibitor and systemic chemotherapy 3. Although hepatocellular carcinoma (HCC) is sensitive to ionizing radiation, conventional radiation plays a limited role in the treatment of HCC because the tolerated dose of the liver is relatively lower 4. Carbon ions (CI) radiation has a sharp energy deposition at the end of its range, known as the Bragg peak, which can increase the dose to the tumor target without increasing toxicity to surrounding non-cancerous tissues and organs, thereby bring better therapeutic efficacy on early and intermediate HCCs 5-7.

Abnormality in the ER signals or quality control mechanisms triggers a stress state named as ER stress 8. Cells alleviate ER stress through unfolded protein response (UPR), which is regulated by three transmembrane sensors, namely PKR-like ER kinase (PERK), activating transcription factor 6 (ATF6) and inositol requiring enzyme 1 (IRE1) 9, 10. Under normal physiological conditions, they bind to immunoglobulin heavy chain binding protein (Bip) and stay inactive 11. Once ER stress is activated, Bip dissociates from the aforementioned transmembrane sensors, and selectively binds to the unfolded or misfolded proteins through its substrate-binding domain. After dissociation, the transmembrane sensors are activated, then a series of corresponding response procedures are initiated to reconstruct ER homeostasis and maintain cell survival 12. However, if the steady-state reconstruction mechanism fails, the signal output of UPR will switch from pro-survival to pro-apoptotic, leading to the activation of the endogenous apoptosis 13.

PERK activation mediates phosphorylation of eIF2α at Ser51, reduces the translation of common proteins and maintains cell survival. PERK also selectively translates the transcription factor ATF4 and regulates the expression of genes related to amino acid biosynthesis, oxidative stress, and apoptosis. PERK can also induce the expression of genes related to radioresistance through Nrf2 phosphorylation 14, 15. Briefly, PERK maintains ER homeostasis and enhances the adaptability to oxidative stress through the PERK-eIF2α-ATF4 and PERK-Nrf2 signal cascades, and promotes cell survival through overexpression of Bcl-2 and Bcl-XL 9, 14.

Persistent or intense ER stress signals can promote PERK-induced cell death. One of the mechanisms is that p53 upregulates PUMA and NOXA and induces p53-dependent apoptosis 16, 17. The activation of PERK can up-regulate CHOP transcription, inhibit the expression of Bcl-2, while promoting the expression of pro-apoptotic BIM, and improve protein synthesis and oxidative stress, and eventually lead to apoptosis 18.

Radiation-induced UPR regulates autophagy-related genes through PERK 19. The induction of autophagy can reduce apoptosis while inhibiting ER stress-related autophagy can promote apoptosis and inhibit tumor progression. PERK-mediated ATF4 activation can up-regulate autophagy-related genes such as LC3, BECN1, Atg3, Atg12 and Atg16L1. Although ER stress and autophagy work independently, they share many common features, including protecting cells by reducing stress and inducing cell death under extreme conditions 20, 21.

The present study was aimed at clarifying the mechanisms underlying the sensitivity of HCC cells to high-LET CI radiation, and then providing a theoretical basis for adopting personalized and precise treatment schemes for HCC based on different *TP53* statuses in clinical treatment.

## Materials and Methods

### Cells and Reagents

Human HCC cell lines (HepG2, Hep3B and PLC/PRF/5) were purchased from the Type Culture Collection of the Chinese Academy of Sciences (Shanghai, China). All cell lines were attached with STR profiling and mycoplasma contamination test reports. HepG2, Hep3B and PLC/PRF/5 have *TP53* wild, null and mutant types, respectively. HepG2 cells were maintained in RPMI-1640 medium (HyClone, US), Hep3B and PLC/PRF/5 cells were maintained in MEM medium (HyClone, US). All cell cultures were supplemented with 10% (v/v) fetal bovine serum (FBS) (Bailing, China), 100 U/mL penicillin and 100 μg/mL streptomycin, and incubated in a humidified incubator at 37ºC with 5% CO_2_.

Chloroquine (CQ) and 4-Phenylbutyric acid (4-PBA) were purchased from Sigma-Aldrich (US). Ferrostatin-1 and Sorafenib were purchased from Selleck (US). Primary antibodies p53, PUMA, NOXA, xCT, PTGS2 and 4-Hydroxynonenal were purchased from Abcam (UK), PERK, ATF4, pBCl-2 and GPX4 were purchased from Cell Signaling Technology (US), β-Actin, Bip, GSK3-beta, DRAM, LC3, SQSTM1 ⁄ p62 and Caspase-3 were purchased from Proteintech (China). Secondary antibodies were all purchased from Proteintech (China).

### Transfection with siRNA

About 2×10^5^ cells were inoculated into a 35mm culture dish overnight, and the *TP53*-siRNA (RIBOBIO, China) was transfected with a riboFECT^TM^ transfection kit according to the manufacturer's operating procedures. 5ul siRNA was added to 120ul riboFECT^TM^ Buffer and mixed gently, then added 12ul riboFECT^TM^ Reagent and mixed by pipetting repeatedly, and incubated at room temperature for 15min to prepare the transfection complex. 863ul of complete medium without penicillin or streptomycin was added into the transfection complex and mixed gently. The prepared mixture was added to the culture dish containing adherent cells, 1ml of culture medium was reserved to make the total volume of the culture solution 2ml. After 48 hours, the medium was replaced and then the cells were irradiated. The target sequence of si-h-*TP53* was *GACTCCAGTGGTAATCTAC*.

### Irradiation

CI: 3×10^5^ cells were inoculated into a 35mm culture dish, when the cell density reaches about 80%, irradiations were carried out with a CI beam of 165 MeV/u in the heavy-ion therapy terminal of the Heavy Ion Research Facility in Lanzhou (HIRFL) at the Institute of Modern Physics, Chinese Academy of Sciences (Lanzhou, China). The LET value of the CI beams was adjusted to 50 keV/ μm on the cell samples.

X-rays: 3×10^5^ cells were inoculated into a 35mm culture dish, when the cell density reaches about 80%, irradiations were performed with an X-ray apparatus (X-Rad225, PRECISION, US) operating at a voltage of 225 kV and a dose rate of 1 Gy/min.

All the irradiations were carried out at room temperature, and the control groups were subjected to sham irradiation.

### Cell survival assay

Cell survival was detected with the colony formation assay as reported previously 22. Cells in the logarithmic growth phase were irradiated with CI for 0, 1, 2, 3, 4, and 6 Gy, respectively. The irradiated cells were seeded into 60mm cell culture dishes, with 3 parallels for each treatment group. After two weeks of culture, the medium was discarded, the adherent cells were stain with crystal violet solution for 30 minutes. The number of clones formed was counted (a clone was identified as a community with more than 50 cells), and the cell survival rate of each culture dish was calculated.

### Cytoplasmic calcium detection

Calcium concentration in the cytoplasm was measured with Fluo-4, AM Fluo Calcium Indicators (Invitrogen, US) according to the manufacturer's instructions. The Fluo-4, AM ester stock solution was diluted to a final working concentration of 5 µM with medium. Cells were incubated with the Fluo-4, AM ester for 30 minutes at 37°C, then washed 3 times with PBS, incubated for another 30 minutes to ensure complete de-esterification of intracellular AM esters, and then detected via fluorescence microscopy or flow cytometry.

### Cell cycle assay

Cells were collected and stained with a commercial kit according to the manufacturer's instructions (Beyotime, China). Cells were digested with EDTA-free trypsin, washed with pre-cooled PBS and collected by centrifugation, then fixed with pre-cooled 70% ethanol at 4°C for 24 hours. Then cells were collected by centrifugation and stained 500ul propidium iodide containing RNase A Dyeing solution, incubated for 30 minutes at 37°C in the dark, and detected by flow cytometry immediately. The cell cycle was analyzed with the Modfit LT software (version 3.1).

### Apoptosis assay

Cells were stained with FITC Annexin V Apoptosis detection kit (BD Pharmingen, US) according to the manufacturer's instructions. Digested Cells were washed with pre-cooled PBS and resuspended in 1ml Binding Buffer, added 5 µl of FITC Annexin V and 5 µl PI, gently vortexed and incubated for 15 minutes at RT in the dark, and apoptosis was analyzed via flow cytometry immediately.

### DNA double-strand breaks

Cells were pretreated and fixed with freshly prepared 4% paraformaldehyde for 10 minutes, and permeabilized with 0.25% Triton-X 100 for 10 minutes. Then, cells were blocked with 5% BSA for 60 minutes, incubated with γH2AX primary antibody (2 hours) and fluorescent secondary antibody (45 minutes) in sequence at room temperature. The slides were stained with Hoechst 33342 and sealed with 20ul anti-quencher and inspected with a fluorescence microscope (OLYMPUS BX51).

### qRT-PCR

Total RNA was extracted using TRIzol reagent (Invitrogen, US) according to the standard procedures 23 and concentration was measured with a microplate reader. cDNA was synthesized with riboSCRIPT Reverse Transcription Kit (RIBOBIO, China) according to the manufacturer's protocol. Quantitative real-time PCR was performed using QuantiNova SYBR Green PCR Kit (QIAGEN, Germany) and reaction samples were run on a QuantStudio5 qPCR system (ThermoFisher, US). The primers of qRT-PCR were synthesized by RIBOBIO (China). The Ct values for each gene were normalized to those of GAPDH, and the 2^-ΔΔCt^ method was used for quantitative analysis of gene expression.

### Immunoblot analysis

The whole proteins were extracted and conducted to SDS-PAGE as the standard procedure described previously 24. The blots were transferred to PVDF membrane, blocked with blocking buffer (Beyotime, China) for 10 min, incubated with primary antibodies at 4 ℃ overnight, then incubated with horseradish peroxidase-conjugated secondary antibodies for 1 hour and finally visualized by the enhanced chemiluminescence (ECL) procedure. The relative expression level of each protein was compared with the individual band density for beta-Actin and normalized by the corresponding control band.

### Statistical analysis

Cell count and measurement of blots density were performed with the ImageJ software (v1.52). Data were presented as mean ± standard deviation (SD). The variance homogeneity test and mean ANOVA analysis was performed with the IBM SPSS software (v21.0). Differences were considered significant and extremely significant when *p*<0.05 (*) and *p*<0.01 (**), respectively.

## Results

### ER stress induced by CI irradiation

**Figure 1:**
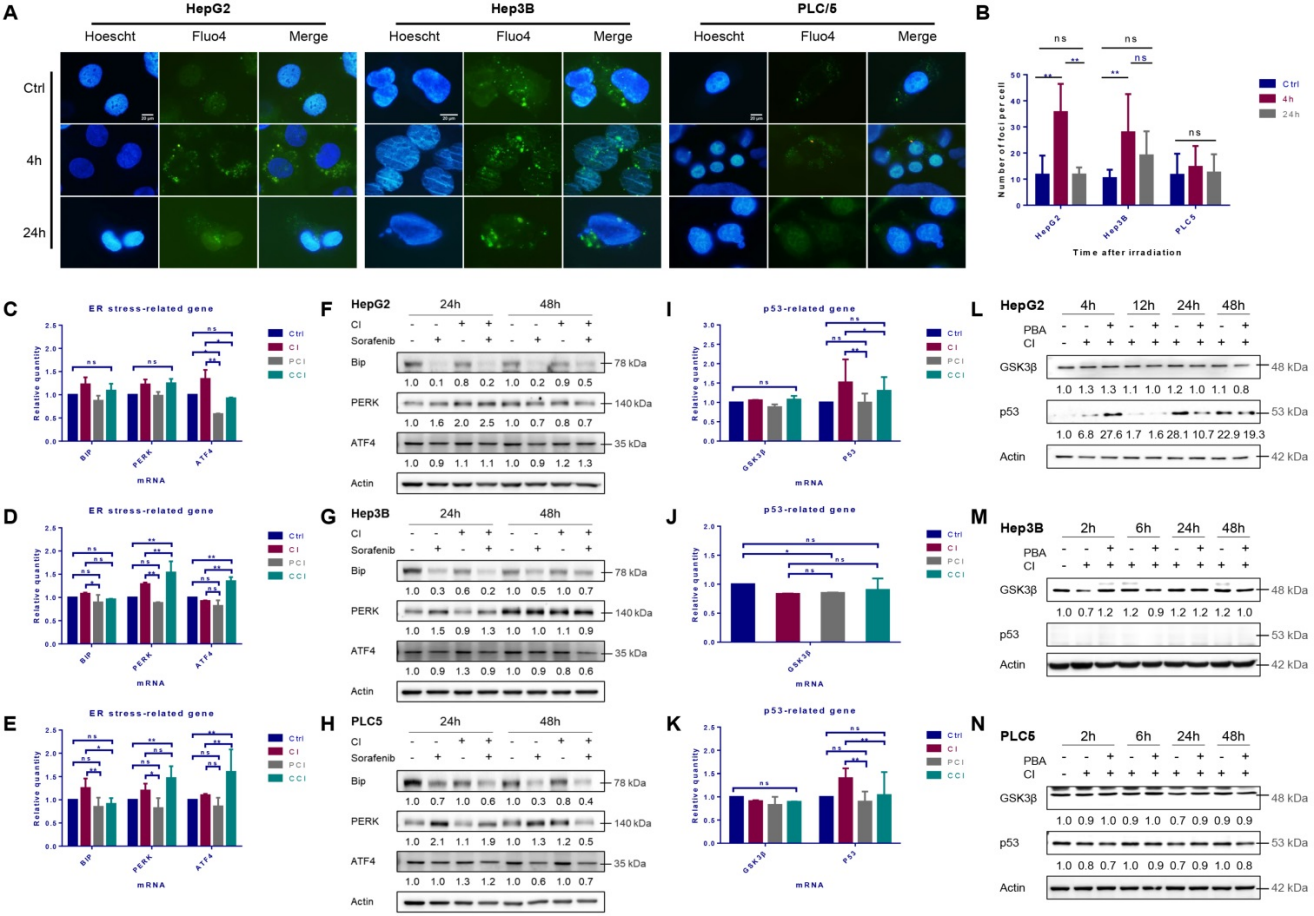
High-LET carbon ions (CI) promoted the expression of p53 and induced ER stress in HCC cell lines. HepG2, Hep3B and PLC/PRF/5 cells were pre-treated with 10 μM CQ for 4h, 0.5 mM 4-PBA for 12h, 10 μM sorafenib for 12h, respectively. Irradiations were conducted with carbon ions (LET = 50 keV/μm) at a dose of 2 Gy. The cytosolic calcium ions (Fluo-4, AM labeled) fluctuated in the three HCC cell lines after irradiation (A, B). The mRNA and protein levels of ER stress-related genes in HepG2 (C, F), Hep3B (D, G) and PLC/PRF/5 (E, H) cells after irradiation. The levels of p53 mRNA and protein in HepG2 (I, L), Hep3B (J, M) and PLC/PRF/5 (K, N) cells after irradiation. (Ctrl: control; PCI: CI treatment combined with 4-PBA; CCI: CI treatment combined with CQ. ns: not significant, *: *p* < 0.05, **: *p* < 0.01).

The cytosolic Ca^2+^ in HepG2 cells increased significantly 4 hours after irradiation with CI and returned to the basal level 24 hours after irradiation. CI irradiation also caused continuous Ca^2+^ release in Hep3B cells and the release maintained until 24 hours after irradiation. However, the CI irradiation had no significant effect on the cytoplasmic Ca^2+^ concentration in PLC/PRF/5 cells (Fig. 1A, B and S1A).

The mRNA levels of Bip in the three HCC cell lines were up-regulated after irradiation, and the levels of PERK and ATF4 mRNAs in the downstream also increased in varying degrees. 4-PBA inhibited the up-regulation of Bip, PERK and ATF4 mRNA levels caused by irradiation (Fig. 1C, D and E). The autophagy inhibitor CQ did not reduce the levels of ER stress-related mRNA significantly.

CI irradiation alone did not change the expression of Bip in the three HCC cell lines, while the expression of PERK was up-regulated in HepG2 and PLC/PRF/5 cells at 24h and 48h, respectively. CI increased ATF4 in HepG2 cells at 48h while in Hep3B and PLC/PRF/5 cells at 24h. CI combined with sorafenib treatment further enhanced the expression of the above genes in HepG2 cells (Fig. 1F, G and H).

CI irradiation up-regulated the mRNA expression level of *TP53* gene in HepG2 and PLC/PRF/5 cells (Fig. 1I and K), and the expression of p53 in HepG2 cells increased significantly post-irradiation, while there was no significant effect on the expression of p53 in PLC/PRF/5 cells and no p53 was detected in Hep3B cells (Fig. 1J, and L-N).

### Autophagy induced by CI irradiation

**Figure 2:**
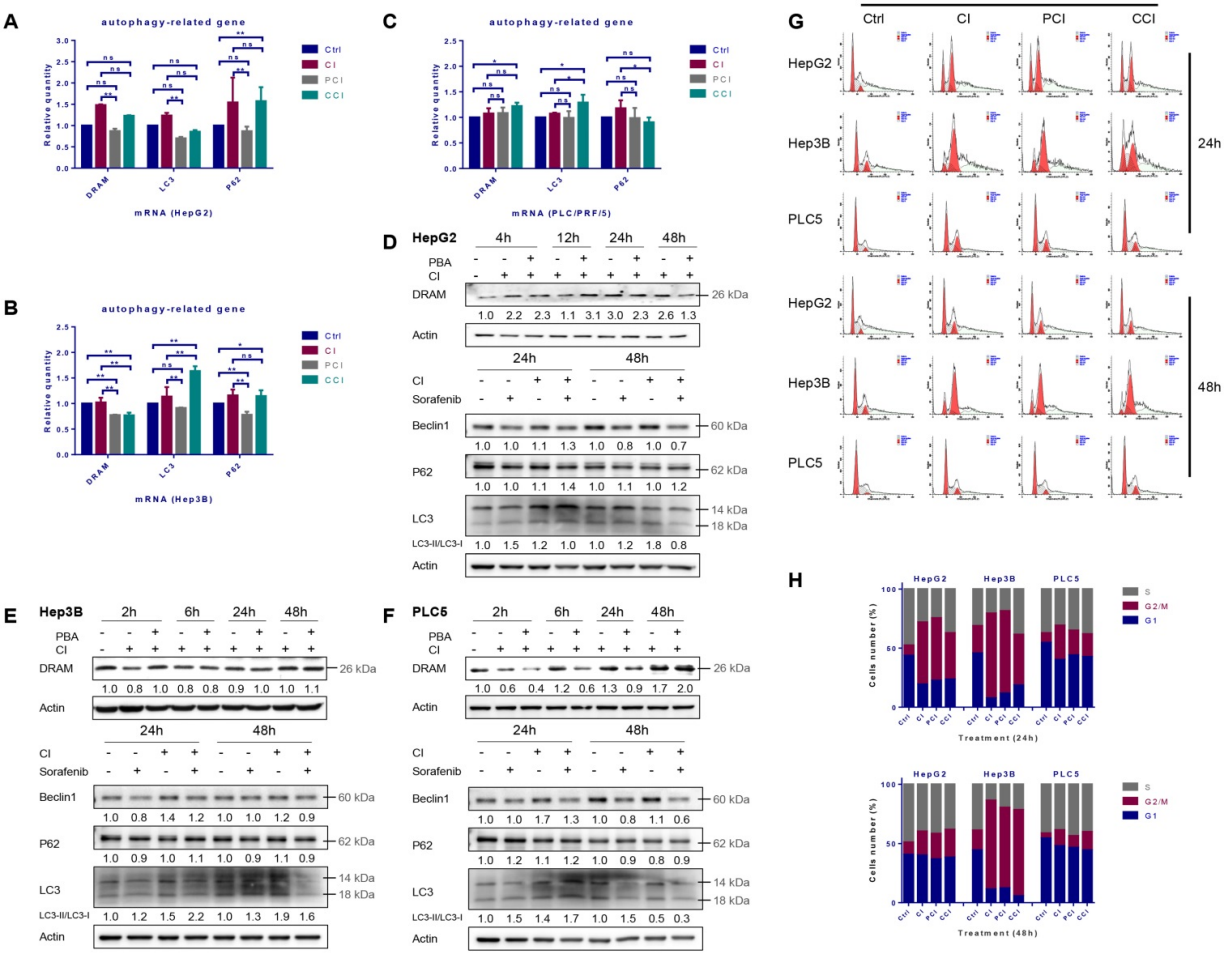
Autophagy and cell cycle arrest induced by carbon ion irradiation. HepG2, Hep3B and PLC/PRF/5 cells were pre-treated with 10 μM CQ for 4h, 0.5 mM 4-PBA for 12h, 10 μM sorafenib for 12h, respectively. Irradiations were conducted with carbon ions (LET = 50 keV/μm) at a dose of 2 Gy. The mRNA and protein levels of autophagy-related genes in HepG2 (A, D), Hep3B (B, E) and PLC/PRF/5 (C, F) cells after irradiation. Cell cycle arrest induced by carbon ion irradiation in the three HCC cell lines (G, H). (Ctrl: control; PCI: CI treatment combined with 4-PBA; CCI: CI treatment combined with CQ. ns: not significant, *: *p* < 0.05, **: *p* < 0.01).

The mRNA levels of DRAM, LC3 and p62 were up-regulated after CI irradiation in all the three HCC cell lines, while 4-PBA combined with CI irradiation reduced the mRNA levels of these autophagy-related genes (Fig. 2A, B and C). The protein levels of DRAM, Beclin-1 and LC3 after irradiation showed a time-dependent trend. Specifically, DRAM and LC3 cleavage increased continuously after irradiation, Beclin-1 increased by CI combined with sorafenib at 24h after treatment in HepG2 cells (Fig. 2D). Irradiation did not augment the expression of DRAM in Hep3B cells, but increased the expression of Beclin-1 and LC3 cleavage at 24h and 48h (Fig. 2E). Moreover, in PLC/PRF/5 cells, CI irradiation induced the up-regulation of DRAM at 48h, while the expression of Beclin-1 and the cleavage of LC3 were enhanced at 24h (Fig. 2F). Notably, p62 increased while LC3 cleavage decreased in HepG2 cells after CI combined with sorafenib treatment. P62 had no significant change in trend after treatment in Hep3B and PLC/PRF/5 cells. In addition, the presence of a double-layer membrane structure containing inclusions in the irradiated cells, namely autophagic vacuoles, was observed with transmission electron microscopy (TEM) (Fig. 4B).

### Cell cycle arrest and apoptosis induced by CI irradiation

Twenty-four hours after irradiation, the G_2_/M phase arrest occurred in all three HCC cell lines. Hep3B cells presented the most significant cell cycle arrest while the level of cell cycle arrest in PLC/PRF/5 cells was the least. Forty-eight hours after irradiation, the cycle arrest of Hep3B cells did not alleviate at all, whereas the cycle arrest recovered to a lower level in HepG2 and PLC/PRF/5 cells (Fig. 2G and H).

**Figure 3:**
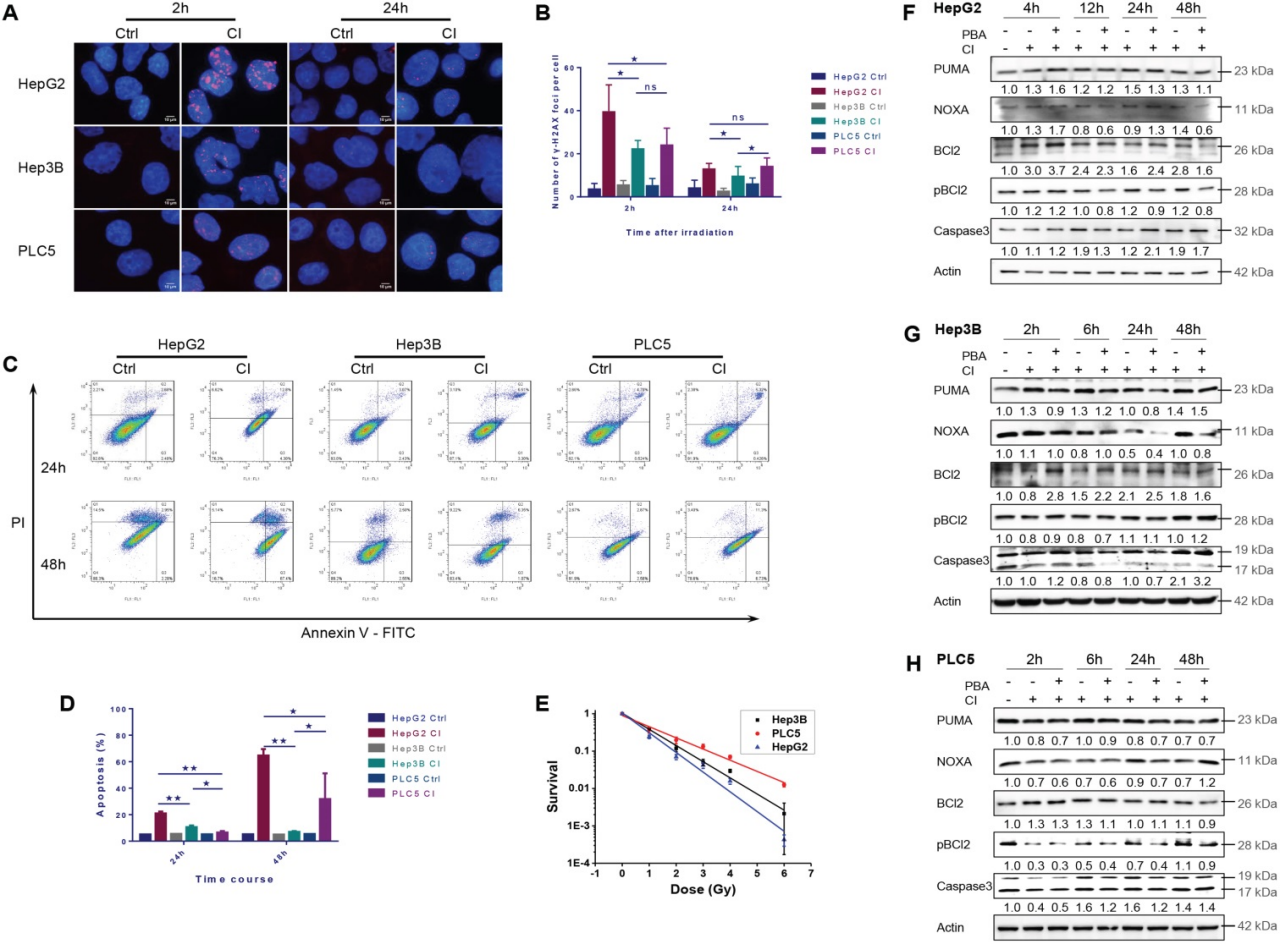
Apoptosis induced by carbon ion irradiation. HepG2, Hep3B and PLC/PRF/5 cells were pre-treated with 0.5 mM 4-PBA for 12h, and then irradiations were conducted with carbon ions (LET = 50 keV/μm) at a dose of 2 Gy. DNA double-strand breaks (Labeled with γH2AX antibody) in three HCCs after irradiation (A, B). The apoptosis rates in the three HCC cell lines after irradiation (C, D). The clonogenic survival data of the three HCC cell lines after irradiation (E). HCC cells were irradiated with carbon ions at a dose of 0, 1, 2, 3, 4 and 6 Gy, respectively. The expression levels of apoptosis-related proteins in HepG2 (F), Hep3B (G) and PLC/PRF/5 (H) after irradiation.

CI irradiation caused DNA DSBs in all three HCC cell lines. The average γH2AX foci number per cell in HepG2 cells was significantly higher than those in Hep3B and PLC/PRF/5 cells at 2 hours after irradiation. Twenty-four hours after irradiation, the average number of foci in all three cell lines decreased, and the number of foci in Hep3B cells was significantly lower than those in the other two cell lines (Fig. 3A and B).

Twenty-four hours after CI irradiation, the apoptotic rate of HepG2 cells was significantly higher than those of the other two cell lines. At 48h after irradiation, the apoptotic rates of HepG2 and PLC/PRF/5 cells increased compared with 24h post-irradiation, while the apoptotic rate of Hep3B cells remained at a low level (Fig. 3C and D).

At the same dose, the cellular sensitivity to CI irradiation became low for *TP53* wild-type HepG2, *TP53*-null Hep3B and *TP53* mutant PLC/PRF/5 cells in turn (Fig. 3E).

CI irradiation up-regulated the expression of PUMA, NOXA and Bcl-2, and PBA inhibited the phosphorylation of Bcl-2 in HepG2 cells. In Hep3B cells, CI irradiation up-regulated the expression of PUMA, down-regulated the level of pBcl-2 2-6h post-irradiation, and PBA inhibited the phosphorylation of Bcl-2, resulting in Caspase-3 cleavage and cellular apoptosis. In PLC/PRF/5 cells, CI irradiation down-regulated the expression of PUMA and NOXA, while PBA inhibited the phosphorylation of Bcl-2, which also increased the cleavage of Caspase-3 and apoptosis 24-48h post-irradiation (Fig. 3F-H).

### Ferroptosis induced by CI irradiation combined with sorafenib treatment

**Figure 4:**
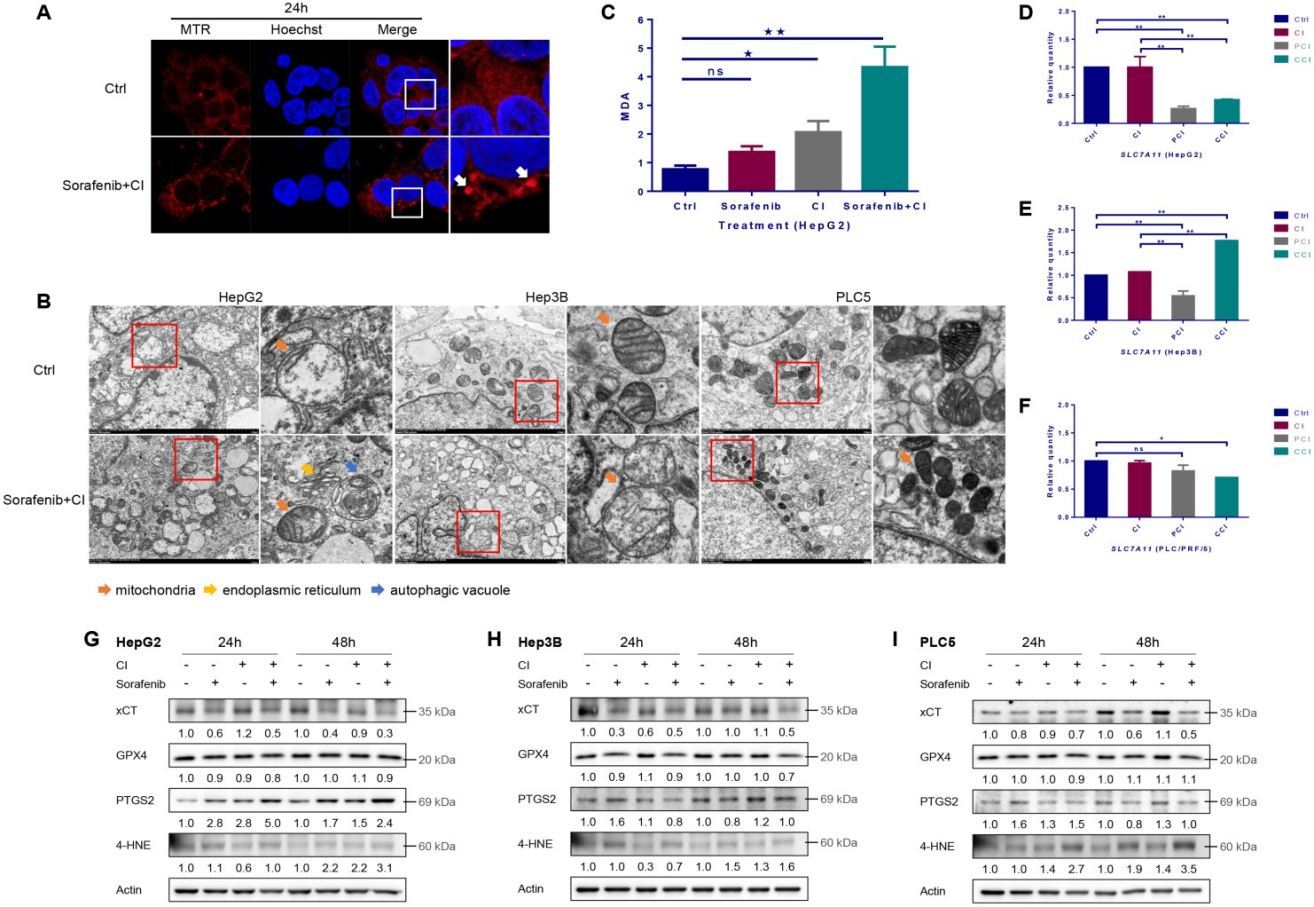
Ferroptosis induced by carbon ion irradiation. HepG2, Hep3B and PLC/PRF/5 cells were pre-treated with 10 μM CQ for 4h, 0.5 mM 4-PBA for 12h, 10 μM sorafenib for 12h, respectively. Irradiations were conducted with carbon ions (LET = 50 keV/μm) at a dose of 2 Gy. (A) Representative morphological characteristics of mitochondria were observed using laser confocal microscopy. (B) Representative characteristics of ferroptosis and autophagic vacuoles were examined with a transmission electron microscope. (C) MDA level in HepG2 cells after carbon ion irradiation. The mRNA levels of *SLC7A11* in HepG2 (D), Hep3B (E) and PLC/PRF/5 (F) cells after irradiation. The expression levels of ferroptosis-related proteins in HepG2 (G), Hep3B (H) and PLC/PRF/5 (I) cells after irradiation. (Ctrl: control. ns: not significant, *: *p* < 0.05, **: *p* < 0.01).

By using Mito-Tracker Red CMXRos probe to label mitochondria, the images of laser scanning confocal microscopy (LSCM) showed that the mitochondria changed from diffuse state into dot state after CI combined with sorafenib treatment, and the fluorescence intensity increased compared with the control group (Fig. 4A). The results of transmission electron microscopy (TEM) showed that CI combined with sorafenib treatment increased mitochondrial membrane density and decreased mitochondrial crest (Fig. 4B). CI irradiation caused an increase in the level of lipid peroxidation product malondialdehyde (MDA), and CI combined with sorafenib treatment further increased the level of MDA significantly (Fig. 4C).

Irradiation had no significant effect on the mRNA level of *SLC7A11* in HepG2 and PLC/PRF/5 cells, but 4-PBA or CQ reduced the mRNA level of *SLC7A11* (Fig. 4D and F). In Hep3B cells, irradiation slightly increased the mRNA level of *SLC7A11*. 4-PBA reduced the mRNA level, but CQ significantly increased the mRNA level of *SLC7A11* (Fig. 4E).

The expression level of xCT (encoded by *SLC7A11*) was down-regulated by sorafenib and further decreased by CI combined with sorafenib treatment in all the three HCCs (Fig. 4G, H and I). The level of GPX4 was slightly down-regulated by CI combined with sorafenib treatment in HepG2 and Hep3B cells, but not in PLC/PRF/5 cells. CI irradiation increased the expression of PTGS2 at 24h and 48h and even the combined treatment further, and increased the expression of 4-HNE at 48h in HepG2 cells. In comparison, CI combined with ferroptosis inhibitor Ferrostatin-1 treatment up-regulated the level of xCT and GPX4, while decreased PTGS2 in HepG2 cells (Fig. S1B). In Hep3B cells, CI irradiation up-regulated the level of PTGS2 and 4-HNE at 48h, while the combined treatment decreased PTGS2 and 4-HNE at 24h but increased 4-HNE at 48h. In PLC/PRF/5 cells, CI irradiation up-regulated the level of PTGS2 and 4-HNE at 24h and 48h. The combined treatment increased PTGS2 at 24h and 4-HNE at 24h and 48h.

### Interference of *TP53* expression affected the radiosensitivity of HepG2 cells

**Figure 5:**
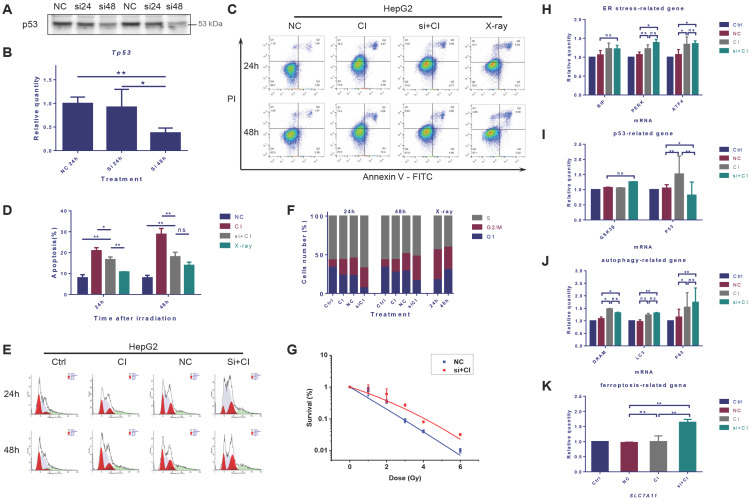
Interference of *TP53* affected the radiosensitivity of HepG2 cells. HepG2 cells were transfected with *TP53*-siRNA for 48h, and irradiations were conducted with carbon ions (2Gy) or X-rays (4Gy). The expression level of *TP53* gene in HepG2 cells after siRNA interference (A, B). The apoptosis rate of HepG2 cells caused by carbon ion irradiation after *TP53* interference (C, D). The cell cycle arrest of HepG2 cells caused by carbon ion irradiation after *TP53* interference (E, F). The clonogenic survival data of HepG2 cells after *TP53* interference (G). In the case of *TP53* interference, the levels of mRNA related to ER stress, p53, autophagy and ferroptosis in HepG2 cells after irradiation (H-K) (Ctrl: control; NC: negative control; si + CI: CI treatment combined with siRNA. ns: not significant, *: *p* < 0.05, **: *p* < 0.01).

Protein expression and mRNA transcription of p53 were significantly reduced 48 hours after *TP53* was inhibited by siRNA (Fig. 5A and B). The cellular apoptosis induced by irradiation after interference was significantly lower than that in the irradiation alone group (Fig. 5C and D). Besides, after the inhibition of *TP53*, CI irradiation caused more significant G_2_/M phase arrest and increased the proportion of S phase cells (Fig. 5E and F). Inhibiting *TP53* reduced the sensitivity of HepG2 cells to CI radiation (Fig. 5G). The interference of *TP53* had no significant effect on the transcription of Bip, but slightly increased the mRNA level of PERK and ATF4. Inhibition of *TP53* also up-regulated the transcription of autophagy-related LC3 and p62, together with the mRNA expression level of the ferroptosis-related gene *SLC7A11* (Fig. 5H-K).

## Discussion

Ca^2+^ fluctuation is considered as a sign of ER damage, and is also one of the characteristic phenomena of ER stress 25. The present study showed that different *TP53* statuses lead to different degrees of Ca^2+^ release caused by CI irradiation. Studies have demonstrated that UPR plays a vital role in the treatment of HCCs by regulating the downstream pathways to favor pro-survival or pro-death signaling 26. The mRNA expression levels of Bip, PERK and ATF4 were up-regulated by CI irradiation in the three HCC cell lines, and the translation of these mRNAs also increased, suggesting that ER stress was induced by high-LET radiation. PERK, often used as a tumor marker, indicates a higher tumor grade and a poor prognosis 27. Inhibiting the activity of PERK can improve the radiosensitivity of tumor cells both *in vitro* and *in vivo* 28, 29. Therefore, PERK might be used as a potential therapeutic target, and the development of new drugs or exploring new mechanisms of existing clinical drugs targeting PERK will have significant therapeutic benefits against cancer.

Autophagy plays a "double-edged sword" role in both cytotoxicity and cytoprotection 30-32. DRAM is a key molecule for p53-activated autophagy. We confirmed that PERK and p53 signals jointly induced autophagy in HepG2 cells. Autophagy was induced by the PERK-ATF4 signaling in Hep3B and PLC/PRF/5 cells, so 4-PBA reduced the mRNA levels of autophagy-related genes in the above two HCC cells. CQ did not reduce the levels of ER stress-related mRNA significantly, indicating that the autophagy signal was in a relatively downstream position of the ER stress signaling network. These results are consistent with the findings that radiation-induced protective autophagy helps to clean the aggregated unfolded proteins, assists the quality control of ER proteins, and finally promotes the survival of tumor cells in the previous studies 19, 30.

The pro-death signal of UPR will eventually converge on the endogenous apoptotic pathway. PUMA and NOXA are two typical Bcl-2 family proteins regulated by p53. By up-regulating the expression of the pro-apoptotic PUMA and NOXA, the Caspase-3 is cleaved, and p53-dependent apoptosis is executed. Compared with HepG2 cells, there were fewer DNA DSBs and lower apoptosis rates in *TP53*-null Hep3B cells. This might be due to the fact that radiation caused a slight up-regulation of PUMA expression and Caspase-3 cleavage. The extent of DNA DSBs and apoptosis in PLC/PRF/5 cells was between the other two HCC cell lines. Probably CI irradiation inhibited the expression of the anti-apoptotic pBcl-2 and promoted Caspase-3 cleavage. The clonogenic survival data were in line with the results above. These results jointly confirmed that the sensitivity of HepG2 cells to high-LET CI radiation was higher than those of the other two HCC cell lines.

The microscopic imaging results revealed that HCCs after CI combined with sorafenib treatment exhibited shrunken mitochondria with increased membrane density and decreased crest, a morphological feature of ferroptosis. CI irradiation caused an increase in the level of lipid peroxidation product MDA, and CI combined with sorafenib treatment further increased the level of MDA significantly. These results indicated that CI irradiation promoted the level of lipid peroxidation and induced the morphological characteristics of ferroptosis in the HCC cell lines.

xCT is the light chain subunit of the cystine-glutamate reverse transport system X_c_^-^, which can transport glutamate to extracellular space and extracellular cystine into cells 33. Inhibiting the X^-^_c_ system reduces intracellular GSH, leading to the accumulation of lipid reactive oxygen species, and ultimately results in ferroptosis, which is a unique cell death mode different from apoptosis and necrosis 34-36. Previous studies have shown that p53 can promote ferroptosis by transcriptionally suppressing *SLC7A11* or promoting SAT1 and GLS2, and can also suppress ferroptosis by directly inhibiting DPP4 activity or inducing CDKN1A/p21 expression 37, 38. Sorafenib is a multi-kinase inhibitor of Raf-1 and B-Raf, which can simultaneously inhibit a variety of intracellular kinases involved in tumor cell signal transduction, angiogenesis and apoptosis, and exert anti-tumor effects. Sorafenib can also induce ferroptosis by inhibiting system X_c_^-^ activity. In this study, CI irradiation alone had a limited effect on the mRNA and protein levels of *SLC7A11* in all the three HCC cell lines, while CI combined with sorafenib treatment significantly reduced the expression of xCT. These results indicated that the combined treatment of CI and sorafenib initiated extrinsic ferroptosis by inhibiting cell membrane transporters 39, 40.

Glutathione peroxidase (GPX4) uses glutathione to convert lipid hydroperoxides into non-toxic lipid alcohols, thereby reducing lipid peroxidation and inhibiting ferroptosis. In this study, we found that CI had no significant effect on GPX4, indicating that GPX4 did not participate in ferroptosis caused by CI irradiation. In other words, CI did not activate ferroptosis through the intrinsic pathway of blockade of intracellular antioxidant enzymes.

PTGS2 serves as another marker of ferroptosis induction in preclinical models. CI irradiation increased the expression of PTGS2 in the three HCC cell lines to varying degrees, and CI combined with sorafenib treatment further increased the PTGS2 level. 4-HNE and MDA are typical products of lipid peroxidation, they usually act as specific ferroptosis-related damage-associated molecular patterns (DAMPs) to induce ferroptosis-specific inflammatory and immune responses 41. We found that the expression of 4-HNE was up-regulated by CI irradiation and further increased by CI combined with sorafenib in all three HCCs. In HepG2 cells, the level of MDA showed the same trend as 4-HNE. These results indicated that CI irradiation combined with sorafenib treatment inhibited the ROS scavenging system X_c_^-^ pathway, thus leading to the accumulation of lipid peroxidation products, which might eventually lead to ferroptosis and help to enhance the inhibitory effect of high-LET CI irradiation on HCC cells.

We found that inhibition of radiation-induced ER stress mainly caused HepG2 cells to undergo mixed-type RCD of both apoptosis and ferroptosis; however, down-regulation of xCT indicated that ferroptosis with a low level of apoptosis might occur in Hep3B and PLC/PRF/5 cells, demonstrating that ER stress could relieve apoptosis and ferroptosis, and promoting cell survival. Previous studies have shown that activation of autophagy is a common event during ferroptosis, but its role is still inconclusive 35, 42. In this study, inhibition of autophagy resulted in a decrease of *SLC7A11* transcription in HepG2 and PLC/PRF/5 cells. This proves that autophagy could alleviate ferroptosis and promote cell survival in *TP53*-wild cells. After CQ treatment, the mRNA level of *SLC7A11* in Hep3B cells increased significantly, indicating that bulk autophagy could promote ferroptosis in *TP53*-null cells.

There have been many studies on the mechanism of *TP53* in regulating radiosensitivity, and it has been generally believed that the absence of *TP53* reduces the sensitivity to conventional radiations such as X-rays. Our results showed that inhibition of *TP53* reduced apoptosis induced by CI irradiation and promoted cell survival. We also found that HepG2 cells showed an easy-to-recover G_2_ / M phase arrest after irradiation, and inhibiting *TP53* led to a more obvious and difficult-to-recover G_2_ / M phase arrest. This is consistent with the phenomenon observed for cell cycle arrest in *TP53*-null Hep3B cells. Interference of *TP53* significantly up-regulated the mRNA expression level of *SLC7A11*, suggesting that p53 might promote ferroptosis by transcriptionally suppressing *SLC7A11.* This is in line with the conclusion that activation of *TP53* significantly reduces the expression of *SLC7A11* and knockout of *TP53* eliminates the inhibitory effect on *SLC7A11* 43-45. Collectively, *TP53* was involved in cell cycle regulation, apoptosis promotion and ferroptosis induction, which jointly regulated the response of HCC cells to ionizing radiation.

**Figure 6:**
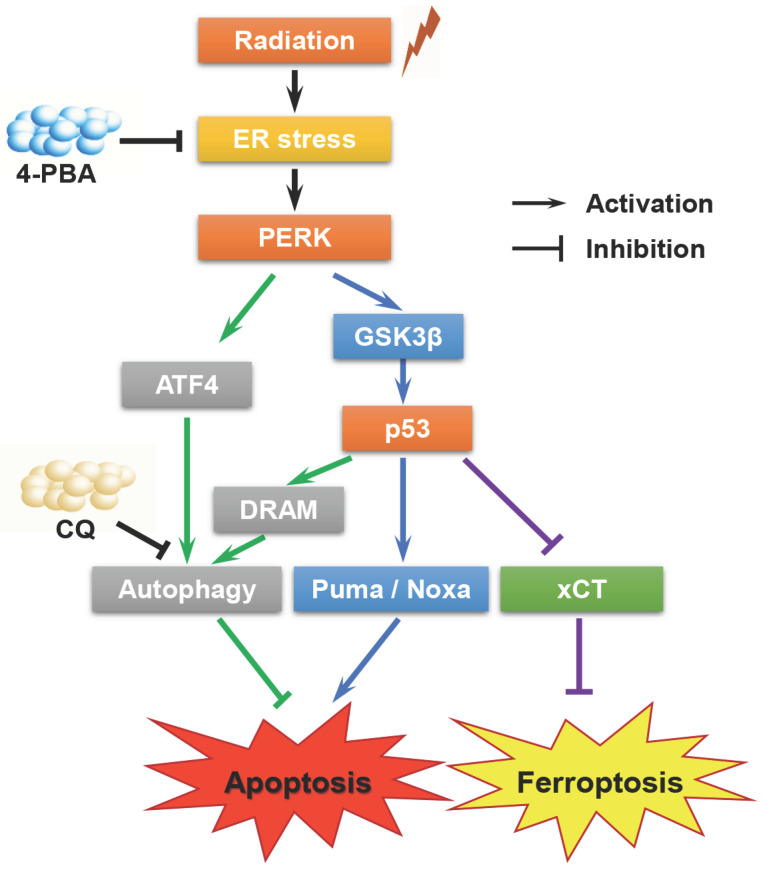
A proposed model of the molecular mechanisms by which PERK regulates the sensitivity of HCC cells with different *TP53* statuses to high-LET carbon ions.

In summary, the present study confirmed that high-LET CI radiation could induce ER stress in HCC cell lines. On the one hand, PERK promoted autophagy and maintained cell survival under ER stress via regulating ATF4 expression; on the other hand, PERK regulated p53 expression, and the latter either induced autophagy by up-regulating DRAM, or directly promoting apoptosis through the mitochondrial pathway or promoting ferroptosis via inhibiting the transcription and translation of *SLC7A11*. Besides, p53 also participated in cell cycle regulation. The above-mentioned factors jointly determined the fate of HCC cells (Fig. 6). Moreover, apoptosis and ferroptosis are synergetic cell death modes regulated by *TP53*. High-LET CI irradiation combined with sorafenib treatment could lead to the induction of a mixed-type RCD including both apoptosis and ferroptosis in HepG2 cells. This might be one of the reasons why the radiosensitivity of *TP53* wild-type HepG2 cells was higher than that of Hep3B and PLC/PRF/5 cells. Due to such a central role of *TP53* in cell signaling, its deletion or mutation directly increases cellular radioresistance. Therefore, our work may shed light on the potential therapeutic implication of high-LET CI radiotherapy combined with PERK targeted drugs to implement personalized and precise treatment of HCCs with different *TP53* statuses.

## Supplementary Material

Supplementary figure.

## Abbreviations

4-PBA: 4-phenylbutyric acid
ATF6: activating transcription factor 6
CI: carbon ions
CQ: chloroquine
ER: endoplasmic reticulum
HCC: hepatocellular carcinoma
IRE1: inositol requiring enzyme 1
LET: linear transfer energy
PERK: PKR-like ER kinase
RCD: regulated cell death
*SLC7A11*: solute carrier family 7 member 11
UPR: unfolded protein response
