# Supplementary material for: PERK Regulates the Sensitivity of Hepatocellular Carcinoma Cells to High-LET Carbon Ions via either Apoptosis or Ferroptosis
Source: J Cancer. 2022 Jan 1;13(2):669–80. doi: 10.7150/jca.61622 (PMC8771512; doi:10.7150/jca.61622)

**Supplementary material**

**Figure S1.** (A) The cytosolic $Ca^{2+}$ (Fluo-4, AM labeled) fluctuated in HCC cells after carbon ion irradiation measured with flow cytometry. Red line: control; light blue line: irradiation with carbon ions at 2Gy; orange line: irradiation with carbon ions at 4Gy. (B) The expression level of ferroptosis-related proteins in HepG2 cells treated with Fer-1 or sorafenib combined with CI irradiation. CI: carbon ions; Fer-1: Ferrostatin-1.

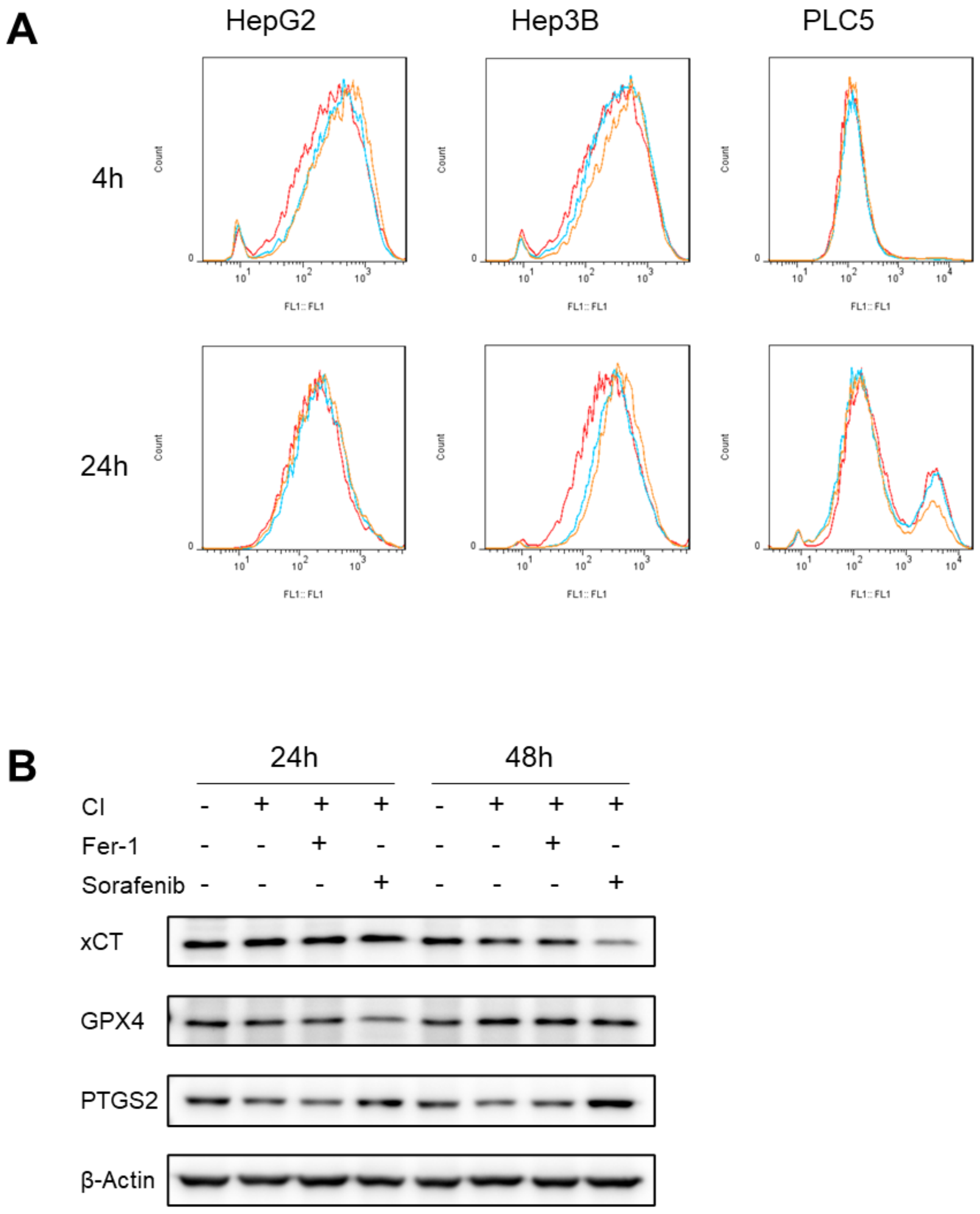

Supplement: Supplementary file 1 — Supplementary figure. [file jcav13p0669s1.pdf]
